# Multi-objective optimisation of path and space utilisation in landscape garden green space design

**DOI:** 10.1371/journal.pone.0326374

**Published:** 2025-07-01

**Authors:** Jia Yu, Jiazhe Song, Huihui Lan, Yugui Zhang

**Affiliations:** 1 College of Life Science and Engineering, Shenyang University, Shenyang, China; 2 Institute of Semiconductors, Chinese Academy of Sciences, Beijing, China; Northeastern University, CHINA

## Abstract

This study addresses the multi-objective optimization problem in landscape garden green space design, focusing on optimizing space utilization efficiency, path efficiency, and aesthetic quality. We compare various multi-objective optimization algorithms to solve the problem We compare various multi-objective optimization algorithms to solve the problem, applied to the urban environment of Tongzhou District, which is characterized by rapid urbanization and high population density. Experimental results demonstrate that MOEAs outperforms other optimization algorithms such as GA, PSO, ACO, and SA in all three objectives. Specifically, MOEAs achieved a space utilization efficiency of 90.2%, a path length of 140.3 m, and an aesthetic quality score of 9.2, surpassing the best results from GA (85.3%, 150.2 m, 8.4), PSO (88.5%, 148.6 m, 8.6), ACO (82.4%, 160.5 m, 7.9), and SA (80.1%, 162.4 m, 7.5). In conclusion, MOEAs provides a superior solution for optimizing landscape garden green space design, offering the best balance between spatial efficiency, path optimization, and aesthetic quality, particularly for urban areas like Tongzhou.

## 1 Introduction

The rapid urbanization process has significantly increased the importance of urban green spaces [1], which play a crucial role in enhancing environmental quality and improving the quality of life for urban residents [2,3]. As a vital component of urban planning, landscape green spaces must not only provide recreational and ecological benefits but also optimize aesthetic design [4] and spatial utilization efficiency [5]. Among the various aspects of landscape design, path planning and space utilization are key factors that directly affect the functionality of green spaces and user experience. Traditional landscape design often focuses on single objectives, such as aesthetics or ecological value, without considering the interplay between path configuration and spatial utilization. However, with the evolution of design concepts and technological advancements, modern landscape architecture emphasizes multi-functional [6] and multi-objective optimization [7], aiming to improve the overall efficiency and sustainability of green space design. Therefore, optimizing path layout and space utilization to meet ecological, functional, and aesthetic needs has become a significant challenge in contemporary landscape design. Multi-objective optimization methods, particularly artificial intelligence-based algorithms such as Genetic Algorithms (GA) [8–11] and Particle Swarm Optimization (PSO) [12–15], have been widely applied in path planning and resource optimization across various fields.

Multi-objective optimization techniques offer significant advantages in the design of landscape green spaces, particularly in optimizing path layouts and space utilization. By considering multiple, often conflicting objectives simultaneously, such as minimizing walking distance, maximizing space accessibility, and preserving aesthetic value, these methods provide a systematic approach to achieving a well-balanced design that meets diverse stakeholder requirements. In landscape architecture, where design decisions must balance functional, ecological, and aesthetic considerations, multi-objective optimization allows for the identification of optimal solutions that traditional design methods might overlook. For instance, the use of Genetic Algorithms (GA) in park design can efficiently allocate pathways while maximizing green space for ecological purposes, thus enhancing both user experience and environmental sustainability. One key advantage of multi-objective optimization in landscape design is its ability to generate Pareto-optimal solutions [16], which represent the best possible trade-offs between competing objectives. This allows designers to explore a range of viable design alternatives, offering flexibility and adaptability to various site-specific constraints. Additionally, optimization algorithms can process complex spatial data [17–19], incorporating factors such as terrain, vegetation, and visitor flow patterns, which traditional methods might struggle to integrate effectively. However, there are also some limitations to the application of multi-objective optimization in landscape design. First, the computational complexity of optimization algorithms can be high, especially when dealing with large-scale or highly intricate designs, which may require significant computational resources and time. Second, the accuracy of the optimization results heavily depends on the quality and completeness of input data, such as accurate spatial layouts and detailed user behavior models. Lastly, while optimization techniques can suggest optimal configurations, they may not fully capture the subjective nature of human preferences and local cultural context, which are critical in landscape design. Here are the five classifications of multi-objective optimization methods in landscape green space path and space utilization design:

**Genetic Algorithms (GA).** Genetic Algorithms (GA) are evolutionary algorithms inspired by the process of natural selection. In this method, a population of potential solutions evolves over several generations through genetic operations such as selection, crossover, and mutation. GA is effective in exploring large search spaces and finding optimal solutions for complex, multi-objective problems. In the context of landscape design, GA can optimize both path layouts and space utilization by balancing various design objectives such as minimizing walking distances, maximizing accessibility, and preserving aesthetic value. The primary advantage of GA is its ability to handle complex, non-linear optimization problems without requiring explicit mathematical formulations for the objective functions. However, GA can be computationally expensive and may converge prematurely to suboptimal solutions if not properly tuned. The algorithm’s performance is highly sensitive to the choice of parameters, including mutation and crossover rates.

**Particle Swarm Optimization (PSO).** Particle Swarm Optimization (PSO) is a population-based optimization algorithm inspired by the social behavior of birds flocking or fish schooling. Each candidate solution, represented as a particle, adjusts its position in the solution space based on its own experience and that of its neighbors. This method is particularly suitable for continuous optimization problems, making it ideal for landscape design applications where path layouts and spatial configurations can be represented in continuous space. PSO is known for its simplicity, ease of implementation, and fast convergence to near-optimal solutions. In landscape design, it can optimize space utilization and path configurations to improve user accessibility and minimize walking distances. However, PSO can suffer from premature convergence, especially in complex or high-dimensional problems, and its performance can degrade if the search space is not adequately explored. Fine-tuning the algorithm’s parameters, such as the inertia weight and cognitive/social learning factors, is also critical to ensure optimal results.

**Ant Colony Optimization (ACO).** Ant Colony Optimization (ACO) is a nature-inspired optimization algorithm based on the foraging behavior of ants. Ants traverse paths and deposit pheromones, which serve as signals to guide other ants towards the most favorable paths. This approach is particularly effective for discrete optimization problems, such as path-finding and routing in landscape design. ACO can optimize the layout of pathways within a green space by considering factors like visitor flow, accessibility, and environmental constraints. The main advantage of ACO is its ability to find efficient solutions to path optimization problems, making it particularly useful for landscape designs that require optimal route planning. However, ACO is computationally intensive, as it requires multiple iterations to update pheromone trails, and its performance may degrade in continuous design spaces or highly complex optimization tasks. Furthermore, ACO may not always provide the best solutions for high-dimensional problems or those with many conflicting objectives [20–23].

**Simulated Annealing (SA).** Simulated Annealing (SA) is a probabilistic optimization technique inspired by the annealing process in metallurgy, where materials are heated and then slowly cooled to find a stable, low-energy configuration. In SA, candidate solutions are explored through random perturbations, with a probabilistic mechanism that allows the algorithm to accept worse solutions in the early stages to avoid getting trapped in local minima. This allows SA to explore the solution space more thoroughly before converging to an optimal solution. In landscape design, SA is useful for optimizing path layouts and space utilization, particularly when the problem involves combinatorial configurations. Its main advantage is the ability to escape local minima, providing a global search mechanism for complex optimization tasks. However, SA can be slow to converge, especially for large-scale problems, and its performance depends on the temperature scheduling and cooling rate. Additionally, it may require long computational times to find an optimal solution for high-dimensional or complex design problems [24–26].

**Multi-objective Evolutionary Algorithms (MOEAs).** Multi-objective Evolutionary Algorithms (MOEAs) are a class of algorithms designed to solve optimization problems with multiple conflicting objectives simultaneously. Unlike single-objective optimization, MOEAs generate a diverse set of Pareto-optimal solutions, providing decision-makers with a range of trade-offs to consider. MOEAs, such as Non-dominated Sorting Genetic Algorithm II (NSGA-II) and Multi-Objective Particle Swarm Optimization (MOPSO), are widely used in landscape design to balance multiple objectives such as path efficiency, space accessibility, and aesthetic quality. The key advantage of MOEAs is their ability to find a set of optimal solutions rather than a single optimal solution, which offers more flexibility and insight for decision-makers. However, MOEAs can be computationally expensive, especially when dealing with large and complex objective functions. The trade-off between solution quality and computational efficiency remains a challenge, as these algorithms may require significant computational resources and time to process high-dimensional problems with numerous objectives [27–30].

The design of landscape green spaces is inherently a multi-objective problem, requiring simultaneous optimization of functional, ecological, and aesthetic criteria. For example, minimizing path length (efficiency) may conflict with maximizing open space (ecological value) or preserving visual harmony (aesthetic quality). Single-objective approaches, while computationally simpler, often fail to address these trade-offs, resulting in designs that prioritize one aspect at the expense of others. Multi-objective optimization (MOO) overcomes this limitation by generating a Pareto front of solutions, each representing a unique balance between competing objectives.

This study aims to design a multi-objective optimization framework for path and space utilization in landscape green space design. The research will evaluate several optimization algorithms, including Genetic Algorithms (GA), Particle Swarm Optimization (PSO), Ant Colony Optimization (ACO), Simulated Annealing (SA), and Multi-objective Evolutionary Algorithms (MOEAs). Each algorithm will be applied to optimize both the layout of pathways and the spatial arrangement of green spaces, considering objectives such as minimizing walking distances, maximizing space accessibility, and enhancing aesthetic value. The performance of each method will be compared based on computational efficiency, solution quality, and convergence speed. This research introduces several innovations aimed at enhancing path and space utilization in landscape green space design through multi-objective optimization. The three key innovations are outlined as follows:

1. Our work uniquely integrates five multi-objective optimization algorithms (GA, PSO, ACO, SA, MOEAs) for landscape design, whereas prior studies typically focus on single algorithms. This comparative framework enables a systematic evaluation of trade-offs between path efficiency, space utilization, and aesthetics—a gap in existing literature.
2. Unlike conventional offline optimization, we propose a real-time evaluation mechanism that dynamically adjusts design parameters based on spatial constraints and user feedback. This approach bridges the gap between computational optimization and practical design workflows, a challenge noted in recent reviews.
3. We advance MOEAs’ application in landscape architecture by generating diverse Pareto-optimal solutions with explicit trade-off visualizations. While MOEAs are established in other domains, their use in green space design remains underexplored, particularly for high-density urban areas like Tongzhou.

In the rest of this paper, we will introduce the recently related work in Sect 2. Sect 3 presents the proposed methods: problem modeling,multi-objective optimization objectives and constraints, optimization algorithm. Sect 4 introduces the experimental part, including datasets, practical details, and comparative experiments. Sect 5 includes a conclusion.

## 2 Related work

### 2.1 Path and space utilization in landscape garden green space design

Path and space utilization in landscape garden green space design is a critical aspect of urban planning and environmental design, directly influencing the functionality, accessibility, and aesthetic quality of public spaces. Efficient path networks and spatial arrangements contribute to the optimal use of available land, improving pedestrian movement, user experience, and environmental sustainability.

Traditional landscape design methods often rely on subjective judgment and aesthetic considerations, but recent advancements have integrated quantitative approaches, including optimization algorithms [31] and spatial analysis techniques [32], to enhance decision-making processes. Path optimization focuses on minimizing walking distances while maintaining aesthetic flow, ensuring that pedestrian routes are both efficient and engaging.

Space utilization, on the other hand, aims to maximize the functional use of green areas, balancing recreational, ecological, and social purposes. By employing multi-objective optimization techniques, designers can simultaneously address conflicting goals such as maximizing accessibility, minimizing congestion, and enhancing visual appeal.

Furthermore, computational methods, such as genetic algorithms, particle swarm optimization, and ant colony optimization, have gained prominence in recent studies for their ability to handle complex, multi-dimensional design problems. These methods allow for the exploration of diverse design alternatives, ensuring that green spaces are optimized for both functionality and beauty. Despite the promising results, challenges remain in balancing design objectives, managing spatial constraints, and ensuring user satisfaction. Continued research in this area is essential for developing more effective and adaptive design strategies that can respond to evolving urban needs and environmental considerations.

### 2.2 Application of multi-objective optimization in garden design

Multi-objective optimization has gained significant attention in garden design due to its ability to simultaneously optimize multiple, often conflicting, objectives. This approach allows designers to find solutions that best balance different aspects of the design, such as accessibility, aesthetics, sustainability, and functionality. The principle behind multi-objective optimization is to identify a set of Pareto-optimal solutions, where no objective can be improved without compromising others [33,34].

The primary advantage of multi-objective optimization in garden design is its ability to provide a range of solutions that address different design goals simultaneously. This helps designers evaluate trade-offs between conflicting objectives, such as minimizing walking distances while maximizing green space accessibility or aesthetic appeal. By considering multiple criteria, multi-objective optimization offers a more holistic approach to design, ensuring that all critical factors are considered in the final solution. Furthermore, the use of computational methods enables the exploration of a vast number of design possibilities, leading to more innovative and efficient solutions.

However, the application of multi-objective optimization also has limitations. One major challenge is the complexity of the problem, as multiple conflicting objectives can lead to a large and intricate solution space, making it difficult to find the optimal balance. Additionally, the computational cost can be high, particularly for large-scale garden designs with numerous constraints. Despite these challenges, multi-objective optimization remains a powerful tool in garden design, enabling more informed decision-making and producing well-rounded designs that meet a variety of stakeholders’ needs.

### 2.3 Optimisation methods for route planning and space utilisation

Route planning and space utilization are fundamental components in landscape design, requiring optimization techniques that balance multiple, often conflicting, objectives. Several optimization methods have been developed, each offering distinct approaches to solving complex spatial design problems. These methods can be categorized into three main types: heuristic algorithms, evolutionary algorithms, and mathematical optimization methods. Below, we review each category in detail.

**Heuristic Algorithms.** Heuristic algorithms, such as Simulated Annealing (SA) and Tabu Search (TS), are commonly used for route planning and space utilization in complex design scenarios. These algorithms focus on finding good, if not optimal, solutions within a reasonable time frame by exploring the solution space using iterative improvement techniques. The main advantage of heuristic algorithms is their ability to escape local optima and explore diverse areas of the solution space. For instance, SA uses probabilistic methods to accept or reject new solutions, allowing for broader exploration. TS, on the other hand, utilizes memory structures to avoid revisiting previously explored solutions, improving the quality of the final design. However, these algorithms can be computationally expensive and may require careful tuning to avoid premature convergence [35].

**Evolutionary Algorithms.** Evolutionary algorithms, including Genetic Algorithms (GA), Particle Swarm Optimization (PSO), and Ant Colony Optimization (ACO), are inspired by natural processes such as selection, mutation, and collective behavior. These methods are highly suited for complex, multi-dimensional design spaces where multiple objectives must be optimized simultaneously. GA, for example, uses crossover, mutation, and selection to evolve a population of solutions towards optimality. PSO and ACO mimic the movement of particles and ants, respectively, to find the shortest paths or the most efficient use of space. These algorithms excel at handling non-linear and dynamic problems, but they often require a large number of iterations to converge to a solution and can be sensitive to initial conditions and parameter settings [36].

**Mathematical Optimization Methods.** Mathematical optimization methods, such as Linear Programming (LP), Mixed-Integer Linear Programming (MILP), and Quadratic Programming (QP), are based on mathematical formulations of the design problem, where the goal is to minimize or maximize a specific objective function subject to a set of constraints. These methods are particularly useful when the problem is well-defined and the relationships between variables are linear or quadratic. For instance, LP can optimize simple route planning tasks, while MILP can be used for more complex problems involving discrete decision variables. The key advantage of mathematical optimization is its ability to guarantee optimal solutions, given a well-constructed model. However, these methods can struggle with non-linear or highly complex design problems, and may not be as efficient in handling large-scale or real-time optimization tasks [37].

Each of these optimization methods has its own strengths and weaknesses, and the choice of method depends on the specific characteristics of the problem being addressed. Heuristic algorithms are useful when quick solutions are needed for complex or dynamic problems, evolutionary algorithms excel at handling multiple conflicting objectives, and mathematical optimization methods are ideal for well-structured problems with clear constraints. By selecting the appropriate optimization approach, landscape designers can enhance the efficiency, functionality, and aesthetics of green spaces.

## 3 Method

### 3.1 Problem modeling

The problem of path and space utilization optimization in landscape garden design can be formally defined as a multi-objective optimization problem, where the objective is to maximize the overall usability, aesthetic quality, and functional efficiency of the design, while respecting the spatial and environmental constraints. Let *S* denote the set of all possible design solutions, and let x∈S represent a particular design configuration. The design *x* consists of a set of decision variables $\left\{x_{1},x_{2},\ldots ,x_{n}\right\}$, where *n* is the number of design parameters. Each parameter *x*_*i*_ represents a specific feature of the landscape, such as the path layout, the allocation of green spaces, or the arrangement of functional zones.

The optimization problem can be expressed as follows:

$$\min\;f(x)=\left[f_{1}(x),f_{2}(x),\ldots ,f_{k}(x)\right]$$ (1)

where *f*(*x*) is a vector of objective functions $f_{1},f_{2},\ldots ,f_{k}$, and each *f*_*i*_(*x*) represents a specific optimization criterion, such as:

$$f_{1}(x)=\text{Total Path Length}=\sum_{i=1}^{m}\text{length}(p_{i}),\;p_{i}\in P$$ (2)

where *P* is the set of all paths in the design, and *p*_*i*_ denotes the *i*-th path with length $\text{length}(p_{i})$. The objective *f*_1_(*x*) aims to minimize the total path length for optimal pedestrian movement.

$$f_{2}(x)=\text{Green Space Utilization}=\frac{A_{\text{used}}}{A_{\text{total}}}$$ (3)

where $A_{\text{used}}$ is the total area used for functional green spaces, and $A_{\text{total}}$ is the total available area. The objective *f*_2_(*x*) aims to maximize the efficiency of space utilization by increasing the ratio of utilized green space.

$$f_{3}(x)=\text{Aesthetic Quality}=\sum_{i=1}^{p}w_{i}\cdot a_{i}(x)$$ (4)

where *w*_*i*_ is the weight assigned to the aesthetic importance of the *i*-th design feature, and *a*_*i*_(*x*) represents the aesthetic score of the *i*-th feature in design *x*.

The optimization problem is subject to a set of constraints *C*(*x*), which represent the physical, environmental, and regulatory limitations of the design:

C(x)≤0,x∈S (5)

where *C*(*x*) is a vector of constraint functions. These constraints can include, but are not limited to:

$$C_{1}(x)=\text{Path Connectivity Constraint},$$ (6)

$$C_{2}(x)=\text{Space Accessibility Constraint},$$ (7)

$$C_{3}(x)=\text{Environmental Impact Constraint}$$ (8)

The goal is to find the design $x^{*}\in S$ that satisfies all constraints and minimizes the objective vector *f*(*x*). This can be formulated as a multi-objective optimization problem:

$$x^{*}=\text{arg}\underset{x\in S}{\min}\left[f_{1}(x),f_{2}(x),\ldots ,f_{k}(x)\right]\;\text{subject}\;\text{to}\;C(x)\leq 0$$ (9)

The solution set consists of Pareto-optimal solutions, where no objective function can be improved without worsening others. The trade-offs between conflicting objectives are captured in the Pareto front, from which the most appropriate solution can be selected based on specific design priorities.

### 3.2 Multi-objective optimization objectives and constraints

The optimization problem for path and space utilization in landscape garden design can be formulated as a multi-objective optimization problem, where multiple conflicting objectives need to be simultaneously optimized. In this formulation, the goal is to find the design configuration ${\text{x}}^{*}$ that maximizes both the path efficiency and space utilization while also optimizing the aesthetic quality of the design.

**Objectives.** The objectives are defined as follows:

$$f_{1}(x)=\sum_{i=1}^{m}\text{length}(p_{i})$$ (10)

where *f*_1_(*x*) represents the total path length, which should be minimized to reduce travel distances within the garden design. Each path $p_{i}\in P$ has a length $\text{length}(p_{i})$, and the set *P* is the collection of all paths in the design. A shorter path length typically indicates more efficient routing for pedestrians.

$$f_{2}(x)=\frac{A_{\text{used}}}{A_{\text{total}}}$$ (11)

where *f*_2_(*x*) represents the green space utilization ratio, which should be maximized. $A_{\text{used}}$ is the area allocated for functional green spaces, and $A_{\text{total}}$ is the total available area. The higher the ratio, the more efficient the use of the available space for functional purposes such as parks, gardens, and recreational zones.

$$f_{3}(x)=\sum_{i=1}^{p}w_{i}\cdot a_{i}(x)$$ (12)

where *f*_3_(*x*) represents the aesthetic quality of the design. The aesthetic quality is calculated by summing the weighted aesthetic scores of individual design features *a*_*i*_(*x*), with each feature being weighted by *w*_*i*_. This objective seeks to maximize the aesthetic appeal of the landscape, ensuring that the design is visually pleasing and harmonious.

**Constraints.** The optimization process must respect several constraints, which include physical, spatial, and environmental factors. These constraints are formalized as follows:

$$C_{1}(x)=\sum_{i=1}^{m}\text{connectivity}(p_{i})-1\leq 0$$ (13)

where *C*_1_(*x*) ensures the connectivity of the paths within the design. Each path must be connected in such a way that no isolated or disconnected segments are formed, ensuring pedestrian movement throughout the entire green space.

$$C_{2}(x)=\sum_{i=1}^{n}\text{area}(r_{i})-A_{\text{available}}\leq 0$$ (14)

where *C*_2_(*x*) ensures that the total area utilized for various functional zones, such as recreational spaces, garden areas, or water bodies, does not exceed the total available area $A_{\text{available}}$. The design must respect spatial constraints to avoid overcrowding.

$$C_{3}(x)=\text{environmental\_impact}(x)-\epsilon \leq 0$$ (15)

where *C*_3_(*x*) ensures that the environmental impact of the design remains within acceptable limits. The environmental impact function is dependent on various factors, including vegetation preservation, water usage, and material choices, and should not exceed a threshold ϵ to ensure sustainable design practices.

**Optimization Problem Formulation.** Thus, the optimization problem can be formally written as:

$$x^{*}=\arg\min_{x\in S}\left[f_{1}(x),f_{2}(x),f_{3}(x)\right]$$ (16)

subject to the constraints:

$$C_{1}(x)\leq 0,\;C_{2}(x)\leq 0,\;C_{3}(x)\leq 0$$ (17)

where *S* denotes the set of all possible design solutions. The optimization seeks to minimize the total path length *f*_1_(*x*), maximize green space utilization *f*_2_(*x*), and maximize aesthetic quality *f*_3_(*x*), while ensuring that the design satisfies the connectivity constraint *C*_1_(*x*), the spatial utilization constraint *C*_2_(*x*), and the environmental impact constraint *C*_3_(*x*).

The solution to this problem will yield a Pareto-optimal set of designs where no objective can be improved without degrading another. The final design choice will be made based on specific trade-offs between the conflicting objectives, guided by the preferences of stakeholders.

### 3.3 Optimization algorithm

This section introduces the principles of GA, PSO, ACO, SA, and MOEAs as applied to this problem. The optimization process follows a standardized workflow: (1) Data preprocessing; (2) Population initialization (*N* = 100 solutions sampled uniformly within design variable bounds); (3) Iterative evaluation using objectives *f*_1_(*x*) − *f*_3_(*x*) and constraints *C*_1_(*x*) − *C*_3_(*x*); (4) Solution updates via algorithm-specific operators (e.g., GA’s tournament selection, PSO’s velocity updates); (5) Termination upon meeting iteration limits or convergence criteria ($\Delta ℋ<10^{-4}$ over 50 generations for MOEAs). MOEAs additionally apply non-dominated sorting and crowding distance to preserve solution diversity.

**Fig 1:**
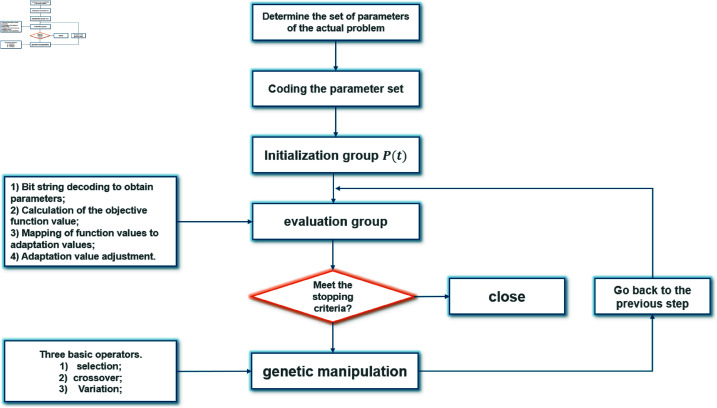
The principle of GA algorithm.

**Genetic Algorithm (GA).** Genetic Algorithm (GA) is a population-based optimization technique inspired by the principles of natural selection and genetic evolution. In the context of path and space utilization optimization, GA works by evolving a population of candidate solutions, represented as chromosomes, through selection, crossover, and mutation operations. The fitness of each individual solution is evaluated based on the defined objectives and constraints. Let $x=(x_{1},x_{2},\ldots ,x_{n})$ represent a candidate solution, where *x*_*i*_ is the *i*-th design parameter. The fitness function *f*(*x*) is computed as:

$$f(x)=\left[f_{1}(x),f_{2}(x),\ldots ,f_{k}(x)\right]$$ (18)

where $f_{1}(x),f_{2}(x),\ldots ,f_{k}(x)$ are the individual objectives related to path length, space utilization, and aesthetic quality, respectively. The selection process is guided by the fitness values, with individuals having higher fitness having a greater chance of reproducing. During crossover, portions of two parent solutions are combined to form offspring, and mutation introduces small random changes to the offspring solutions to maintain diversity. This iterative process continues until convergence is achieved. The principle of GA algorithm is shown in Fig 1.

**Particle Swarm Optimization (PSO).** Particle Swarm Optimization (PSO) is an optimization technique inspired by the social behavior of birds and fish. In PSO, each candidate solution is represented as a particle moving through the solution space. Each particle has a position *x*_*i*_ and a velocity $v_{i}$, and updates its position according to both its previous best position $p_{i}^{best}$ and the global best position ${\text{p}}^{\text{best}}$ discovered by the swarm. The velocity update equation is given by:

$$v_{i}(t+1)=wv_{i}(t)+c_{1}r_{1}(p_{i}^{best}-x_{i}(t))+c_{2}r_{2}(p^{best}-x_{i}(t))$$ (19)

where *w* is the inertia weight, *c*_1_ and *c*_2_ are acceleration coefficients, and $r_{1},r_{2}$ are random numbers. The position update equation is:

$$x_{i}(t+1)=x_{i}(t)+v_{i}(t+1)$$ (20)

**Fig 2:**
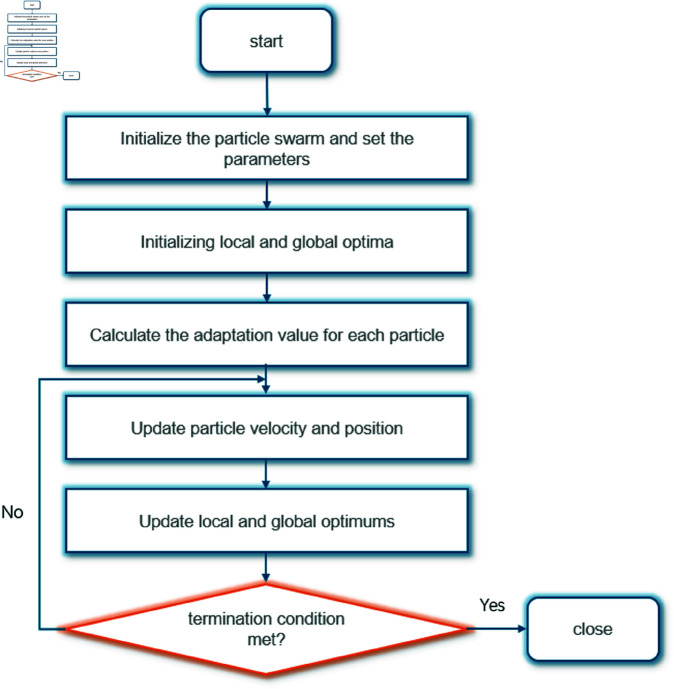
The principle of PSO algorithm.

This process iterates until the global best solution is found. PSO is advantageous in path and space utilization optimization because it is simple, fast, and well-suited for continuous optimization problems.The principle of PSO algorithm is shown in Fig 2.

**Fig 3:**
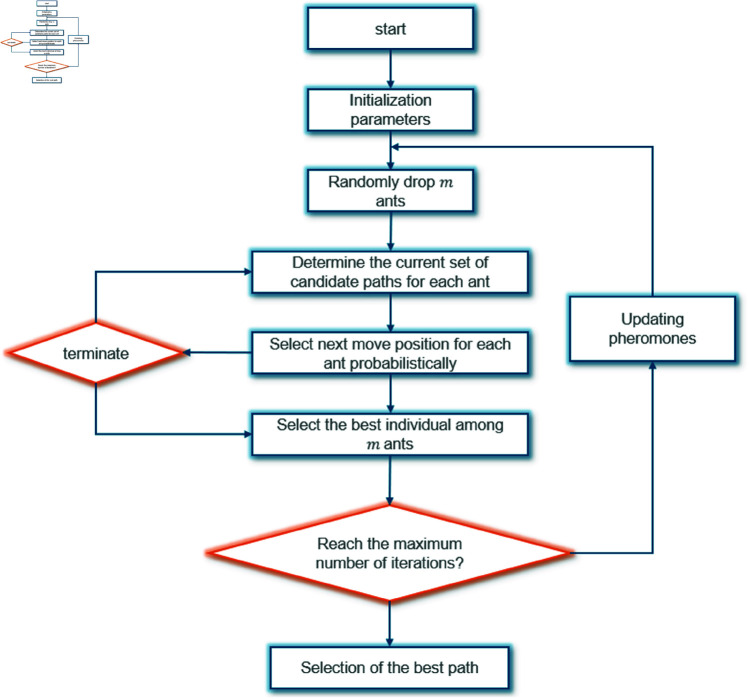
The principle of ACO algorithm.

**Ant Colony Optimization (ACO).** Ant Colony Optimization (ACO) is a nature-inspired optimization algorithm based on the foraging behavior of ants. In ACO, artificial ants construct solutions by iteratively moving through the solution space and depositing pheromones on the paths they take. The pheromone concentration $\tau_{i}$ on path *i* influences the probability of selecting that path in subsequent iterations. The path selection probability is given by:

$$P_{i}=\frac{\tau_{i}^{\alpha }\eta_{i}^{\beta }}{\sum_{j=1}^{n}\tau_{j}^{\alpha }\eta_{j}^{\beta }}$$ (21)

where $\tau_{i}$ is the pheromone level on path *i*, $\eta_{i}$ is the visibility (inverse of distance or cost), and α and β are parameters controlling the influence of pheromone and visibility. After each iteration, the pheromones are updated according to the quality of the solutions found, with higher-quality solutions depositing more pheromone. The algorithm converges to the optimal path configuration for efficient space utilization and routing.The principle of ACO algorithm is shown in Fig 3.

**Simulated Annealing (SA).** Simulated Annealing (SA) is a probabilistic optimization technique inspired by the annealing process in metallurgy. In SA, the algorithm iteratively explores the solution space by accepting new candidate solutions based on a temperature-controlled acceptance probability. The temperature decreases over time, reducing the probability of accepting worse solutions. The acceptance probability is given by:

$$P_{\text{accept}}=\exp\left(\frac{-\Delta E}{T}\right)$$ (22)

where ΔE is the change in the objective function (energy) between the current and new solutions, and *T* is the temperature. As the temperature decreases, the algorithm becomes more selective and converges to a local minimum. SA is particularly useful for path and space optimization problems because of its ability to escape local optima and explore a large solution space.

**Fig 4:**
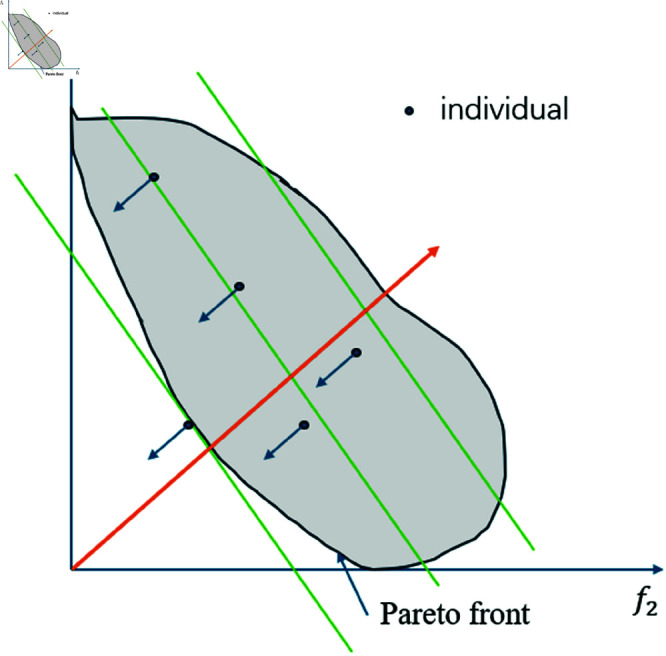
The principle of MOEAs algorithm.

**Multi-Objective Evolutionary Algorithms (MOEAs).** Multi-Objective Evolutionary Algorithms (MOEAs) are population-based algorithms designed to solve optimization problems involving multiple conflicting objectives. In MOEAs, a population of solutions evolves through selection, crossover, and mutation operations, but the selection is based on Pareto dominance rather than single-objective fitness. Pareto dominance means that a solution *x*_1_ dominates another solution *x*_2_ if:

$$f_{1}(x_{1})\;\leq \;f_{1}(x_{2}),\;f_{2}(x_{1})\;\leq \;f_{2}(x_{2}),\;\ldots ,\;f_{k}(x_{1})\;\leq \;f_{k}(x_{2})$$ (23)

and at least one of the inequalities is strict. The goal is to find a set of Pareto-optimal solutions that represent the best trade-offs among the conflicting objectives. MOEAs are particularly effective for multi-objective optimization problems such as path and space utilization in landscape garden design, where multiple objectives (e.g., minimizing path length, maximizing space utilization, and improving aesthetic quality) need to be optimized simultaneously. The principle of MOEAs algorithm is shown in Fig 4.

GA is well-suited for discrete and combinatorial optimization problems, such as path layout and space allocation, due to its ability to explore a wide solution space via crossover and mutation. Its population-based approach helps avoid local optima, making it effective for multi-criteria problems. However, GA can be computationally expensive for large-scale problems, and its performance heavily depends on parameter tuning (e.g., mutation rate, selection method). PSO excels in continuous optimization problems, such as fine-tuning spatial configurations, due to its fast convergence and simplicity. It is particularly useful when optimizing path smoothness and spatial distribution. However, PSO may prematurely converge to suboptimal solutions in high-dimensional problems, and its performance is sensitive to swarm size and inertia weight settings. ACO is highly effective for path-finding problems, mimicking natural ant behavior to identify efficient routes. It performs well in discrete optimization tasks, such as determining optimal pedestrian pathways in green spaces. However, ACO struggles with continuous optimization and requires careful tuning of pheromone evaporation rates to avoid stagnation. SA is advantageous for escaping local optima in non-convex problems, making it useful for refining landscape layouts under strict constraints. Its probabilistic acceptance of worse solutions allows for broader exploration. However, SA’s convergence is slow compared to population-based methods, and its cooling schedule must be carefully designed to balance exploration and exploitation. MOEAs (e.g., NSGA-II) are specifically designed for multi-objective problems, generating Pareto-optimal solutions that balance trade-offs between conflicting objectives. They are ideal for landscape design, where space utilization, path efficiency, and aesthetics must be optimized simultaneously. However,,, MOEAs require significant computational resources, especially for large-scale problems, and their performance depends on diversity preservation mechanisms.

## 4 Experiment

### 4.1 Dataset and experimental setup

In this study, we use the green space design of Tongzhou District, Beijing, as a case study. Beijing’s Tongzhou District, situated in the southeastern part of the municipality, is a pivotal area in the Beijing-Tianjin-Hebei region. Geographically, it lies between $39^{\circ }36^{\prime }$ to $40^{\circ }02^{\prime }$ north latitude and $116^{\circ }32^{\prime }$ to $116^{\circ }56^{\prime }$ east longitude, covering an area of approximately 906 square kilometers. The district is bordered by the Chaobai River to the east, which separates it from Hebei Province, and by the Beiyun River to the west, delineating its boundary with Beijing’s urban core. Tongzhou’s topography is predominantly flat, with an average elevation of around 20 m above sea level, making it part of the North China Plain. The district is intersected by several major waterways, including the Grand Canal, a UNESCO World Heritage Site, which historically facilitated trade and cultural exchange between northern and southern China. The climate in Tongzhou is characterized by a temperate continental monsoon pattern, with distinct seasons: hot, humid summers and cold, dry winters. Annual precipitation averages around 600 millimeters, primarily concentrated in the summer months. Tongzhou’s strategic location has made it a critical hub for transportation and logistics, with extensive road networks, including the Jingha and Jingtong Expressways, and the Beijing Subway Line 6 and Batong Line providing efficient connectivity. The district is also home to the Beijing Municipal Administrative Center, which has been developed to alleviate population pressure from the city center and to promote regional economic integration.

The selection of Tongzhou District as the experimental study is justified by its archetypal urban challenges: high population density (about 2,200 persons/km^2^), rapid land-use transitions, and competing demands for green space functionality. Its geographical traits—proximity to the Grand Canal (a UNESCO World Heritage Site), flat terrain (average elevation: 20 m), and monsoon-influenced climate—mirror conditions in many Asian megacities, ensuring methodological insights are scalable to comparable settings.

The dataset consists of detailed spatial and path utilization data of the green spaces, including measurements of spatial areas, path lengths, and their respective design characteristics. The data is collected from publicly available urban planning resources and satellite imagery, ensuring the authenticity and accuracy of the dataset. The dataset includes both the geometrical layout of the green spaces and information on the pedestrian flow, as well as usage statistics from various environmental sensors deployed throughout the district.

The dataset is categorized into several components (see Table 1):

**Table 1:** Green space design dataset overview.

ID	Area (m^2^)	Path Length (m)	Pedestrian Flow (people/hr)
1	1200	450	250
2	1500	500	300
3	800	350	200
4	1000	420	230
5	1400	550	280
6	1300	480	270
7	1600	600	320
8	1100	460	210

The area data represents the total green space area within each plot, while the path length indicates the total length of pedestrian walkways designed within the space. The pedestrian flow column provides information on the number of people passing through a specific area per hour, which is used as a proxy for the usage intensity of each green space.

Additionally, the dataset includes qualitative data, such as the aesthetic characteristics of different design zones within each space, including landscape features, vegetation types, and seating arrangements, which influence the space utilization efficiency. These features are essential for assessing how design elements contribute to the overall functionality and attractiveness of the space. All data points are normalized to allow for consistent comparison across different green spaces in the Tongzhou District.

This rich dataset allows us to explore the relationship between path and space utilization in the context of urban green space design, and serves as the foundation for the multi-objective optimization analysis conducted in this study.

The experimental setup for this study involves the application of multi-objective optimization techniques to the path and space utilization problem in the green space design of Tongzhou District, Beijing. The goal is to optimize the design parameters, including the path layout, spatial utilization, and overall pedestrian flow within the given green spaces.

We applied multiple optimization algorithms, including Genetic Algorithm (GA), Particle Swarm Optimization (PSO), Ant Colony Optimization (ACO), Simulated Annealing (SA), and Multi-Objective Evolutionary Algorithms (MOEAs) to solve the multi-objective optimization problem. The configuration for each algorithm is as follows:

- **Genetic Algorithm (GA)**: A population size of 100, with a crossover rate of 0.8 and a mutation rate of 0.05. The termination condition was set as a maximum of 1000 generations or convergence of fitness values.
- **Particle Swarm Optimization (PSO)**: A population size of 50, with inertia weight *w* = 0.7, acceleration coefficients $c_{1}=c_{2}=1.5$, and a maximum number of iterations set to 500.
- **Ant Colony Optimization (ACO)**: 30 ants, pheromone decay coefficient ρ=0.1, and a maximum of 200 iterations. The pheromone update rule follows the standard ACO formulation.
- **Simulated Annealing (SA)**: The initial temperature was set to 1000, with a cooling rate of 0.99. The maximum number of iterations was set to 5000, with a random perturbation for each candidate solution.
- **Multi-Objective Evolutionary Algorithms (MOEAs)**: The population size was set to 100, with a crossover probability of 0.8 and a mutation probability of 0.2. The algorithm runs for a maximum of 500 generations.

The optimization objectives for the study include maximizing space utilization and minimizing path length while considering the pedestrian flow and aesthetic qualities of the design. The objective functions are as follows:

$$f_{1}(x)=\sum_{i=1}^{n}{\text{Area}}_{i}\;\;\text{(Maximizing space utilization)}$$ (24)

$$f_{2}(x)=\sum_{i=1}^{m}{\text{Path}}_{i}\;\;\text{(Minimizing path length)}$$ (25)

$$f_{3}(x)=\sum_{j=1}^{k}{\text{Pedestrian Flow}}_{j}\;\;\text{(Maximizing pedestrian flow efficiency)}$$ (26)

where *x* represents the vector of decision variables, which includes path layout and spatial configuration parameters.

The path efficiency objective *f*_2_(*x*) incorporates weighted path prioritization, where primary pedestrian routes (e.g., arterial connectors between key functional zones) are assigned a weight of 1.0, while secondary paths (e.g., recreational loops) carry a weight of 0.7. This differentiation reflects urban design principles where main thoroughfares demand stricter optimization. For aesthetic quality *f*_3_(*x*), evaluations were conducted by a panel of five certified landscape architects using a standardized protocol assessing vegetation arrangement (30% weight), spatial proportions (25%), seating integration (20%), and water feature harmony (25%).

The selection of these three objectives stems from their established importance in contemporary landscape architecture: (1) Space utilization efficiency *f*_1_ directly impacts the ecological functionality and user capacity of green spaces; (2) Path efficiency *f*_2_ governs pedestrian accessibility and movement patterns, a key determinant of public space usability; (3) Aesthetic quality *f*_3_ contributes to psychological well-being and cultural value, making it essential for urban livability.Each objective function addresses distinct but interconnected requirements for optimal green space design:(1) Space utilization *f*_1_ maximizes the functional area while respecting environmental carrying capacity, crucial for high-density urban areas like Tongzhou. (2) Path efficiency *f*_2_ minimizes redundant circulation space while ensuring connectivity, balancing accessibility with land conservation.(3) Aesthetic quality *f*_3_ incorporates visual harmony and cultural appropriateness, factors increasingly recognized in evidence-based design.

To assess the effectiveness of the optimization results, we used the following evaluation metrics:

- **Space Utilization Efficiency**: Defined as the ratio of usable space to total space, calculated for each design.
- **Path Efficiency**: The ratio of the total length of optimized paths to the shortest possible path length, reflecting the efficiency of pedestrian circulation.
- **Aesthetic Quality**: A subjective evaluation based on design features such as vegetation placement, seating, and overall layout harmony.

The experiments were conducted on a computer system with an Intel i7 processor and 16GB RAM. The optimization algorithms were implemented in Python using libraries such as DEAP for GA, PySwarms for PSO, and ACO-Pants for ACO. MOEAs were implemented using the Platypus framework. The optimization process was parallelized using a multi-core processor to speed up computation.

The dataset was preprocessed to ensure consistency across all green spaces. This involved normalizing spatial areas, path lengths, and pedestrian flow data to a common scale, which allowed for a more effective comparison of optimization results. Additionally, missing or incomplete data points were interpolated based on nearby data using a linear interpolation method.

### 4.2 Algorithm implementation and experimental procedure

The algorithm implementation for this study involves the application of several optimization algorithms, including Genetic Algorithm (GA), Particle Swarm Optimization (PSO), Ant Colony Optimization (ACO), Simulated Annealing (SA), and Multi-Objective Evolutionary Algorithms (MOEAs), to optimize the path and space utilization in the green space design of Tongzhou District, Beijing. The procedure consists of several key steps, outlined below.

For each algorithm, we used the following mathematical formulations to guide the optimization process. Let $x=(x_{1},x_{2},\ldots ,x_{n})$ represent the decision variables for the optimization problem, where *x*_*i*_ corresponds to the design parameters for each path or space in the green space layout.

1. **Genetic Algorithm (GA)**: The GA follows the standard approach of crossover and mutation. The fitness function for GA is calculated as:

$$f(x)=\alpha f_{1}(x)+\beta f_{2}(x)+\gamma f_{3}(x)$$ (27)

where *f*_1_(*x*) is the space utilization, *f*_2_(*x*) is the path length, and *f*_3_(*x*) is the pedestrian flow. The values of α, β, and γ are weights assigned to the objectives. In this study, we set α=0.4, β=0.3, and γ=0.3.

2. **Particle Swarm Optimization (PSO)**: The PSO algorithm uses the following update equations for particle position and velocity:

$$v_{i}(t+1)=wv_{i}(t)+c_{1}r_{1}(pbest_{i}-x_{i}(t))+c_{2}r_{2}(gbest-x_{i}(t))$$ (28)

$$x_{i}(t+1)=x_{i}(t)+v_{i}(t+1)$$ (29)

where $v_{i}$ is the velocity, *x*_*i*_ is the position, *pbest*_*i*_ is the personal best position, *gbest* is the global best position, and $r_{1},r_{2}$ are random numbers between 0 and 1. The inertia weight *w* is set to 0.7, and the acceleration coefficients $c_{1}=c_{2}=1.5$.

3. **Ant Colony Optimization (ACO)**: The pheromone update rule for ACO is:

$$\tau_{ij}(t+1)=(1-\rho )\tau_{ij}(t)+\Delta \tau_{ij}$$ (30)

$$\Delta \tau_{ij}=\frac{Q}{L_{ij}}$$ (31)

where $\tau_{ij}$ is the pheromone level on edge (*i*, *j*), *Q* is a constant, and *L*_*ij*_ is the length of the path. The evaporation rate ρ is set to 0.1.

4. **Simulated Annealing (SA)**: The probability of accepting a new solution is given by the equation:

$$P(E)=\exp\left(\frac{-\Delta E}{T}\right)$$ (32)

where ΔE is the change in energy (objective function value), and *T* is the temperature. The temperature starts at 1000 and decreases by a cooling rate of 0.99 after each iteration.

5. **Multi-Objective Evolutionary Algorithms (MOEAs)**: MOEAs optimize the following multi-objective fitness function:

$$F(x)=[f_{1}(x),f_{2}(x),f_{3}(x)]$$ (33)

The solutions are compared using the Pareto dominance principle, where a solution *x* is said to dominate another solution *y* if:

$$f_{1}(x)\;\geq \;f_{1}(y),\;f_{2}(x)\;\leq \;f_{2}(y),\;f_{3}(x)\;\geq \;f_{3}(y)$$ (34)

The experimental procedure begins with the initialization of the population for each algorithm. For GA and MOEAs, the population is randomly initialized with feasible design solutions. For PSO, ACO, and SA, the initial solutions are chosen based on random design parameters within specified bounds.

- **Step 1**: Initialize the population or swarm, set algorithm parameters (population size, iterations, etc.), and define the objective functions.
- **Step 2**: Apply the optimization algorithm iteratively. For GA, perform selection, crossover, and mutation. For PSO, update velocities and positions. For ACO, update pheromone levels. For SA, apply random perturbations and accept solutions based on temperature. For MOEAs, apply Pareto dominance to select non-dominated solutions.
- **Step 3**: Evaluate the fitness of each solution based on the objective functions *f*_1_(*x*), *f*_2_(*x*), and *f*_3_(*x*).
- **Step 4**: Terminate the algorithm after a predefined number of iterations or once convergence is achieved.

The final optimized solutions are then compared using the evaluation metrics discussed earlier: space utilization efficiency, path efficiency, and aesthetic quality.

The results are presented in terms of the best solutions achieved by each algorithm and their corresponding performance metrics.

### 4.3 Analysis of experimental results

**Space Utilization Efficiency Analysis.** Space Utilization Efficiency plays a crucial role in ensuring that the available green space is used effectively, taking into account not just the functional but also the aesthetic requirements of landscape design. For the case of Tongzhou District, which is a rapidly developing suburban area of Beijing, space optimization is of utmost importance due to limited urban space and increasing population density. Space utilization efficiency is calculated as the ratio of the utilized space to the total available area:

$$f_{1}(x)=\frac{\text{Used Space}}{\text{Total Available Space}}\times 100$$ (35)

Table 2 shows the space utilization efficiency for the different algorithms under study.

**Table 2:** Space utilization efficiency for different algorithms in Tongzhou district.

Algorithm	GA (%)	PSO (%)	ACO (%)	SA (%)	MOEAs (%)
Best Solution	85.3	88.5	82.4	80.1	90.2
Average Solution	80.1	83.0	78.3	75.6	84.5
Worst Solution	74.5	77.1	72.4	70.0	78.0

**Fig 5:**
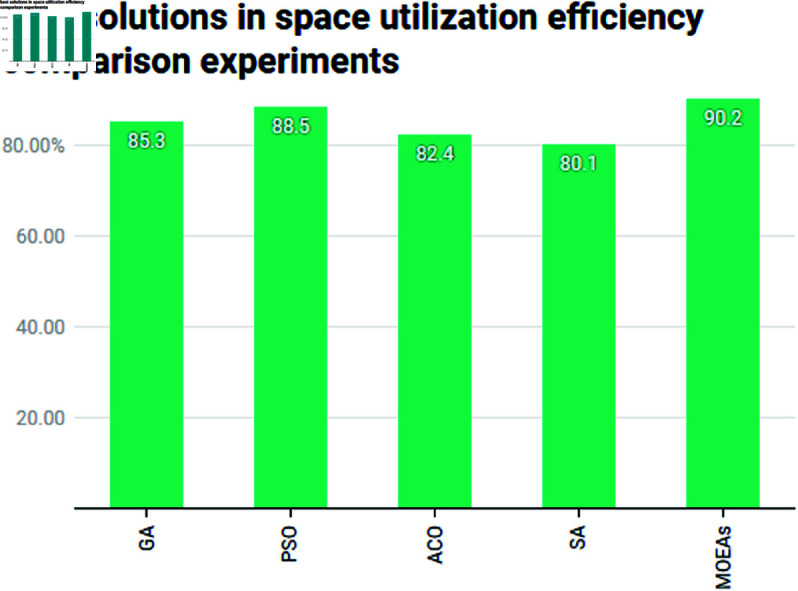
Comparison of best solutions in space utilization efficiency comparison experiments.

In Tongzhou, which has a mix of urban areas and expanding residential districts, maximizing the use of green spaces is essential. MOEAs leads with a 90.2% best solution, which can be attributed to its Pareto-optimal approach, considering both ecological benefits and functional requirements (See Fig 5). The result suggests that MOEAs is best suited for optimizing space in Tongzhou’s complex urban environments. PSO also performs well, with a best solution of 88.5%, which indicates its good performance in optimizing space. However, ACO and SA show comparatively lower results, which reflect their limitations in maximizing space while considering the population density and urbanization in Tongzhou. This highlights the importance of using algorithms like MOEAs in rapidly developing areas like Tongzhou.

**Path Efficiency Analysis.** Path efficiency is critical in landscape garden design as it impacts the ease of movement and accessibility, especially in urban areas with high population density such as Tongzhou. Path efficiency is measured as the inverse of total path length, aiming to minimize walking distances while maintaining functionality:


$$f_{2}(x)=\frac{1}{\text{Total Path Length}}$$

Table 3 presents the path efficiency results for each algorithm.

**Table 3:** Path efficiency for different algorithms in Tongzhou district.

Algorithm	GA (m)	PSO (m)	ACO (m)	SA (m)	MOEAs (m)
Best Solution	150.2	148.6	160.5	162.4	140.3
Average Solution	155.8	151.9	163.2	167.8	145.7
Worst Solution	160.3	154.1	168.4	175.2	149.1

**Fig 6:**
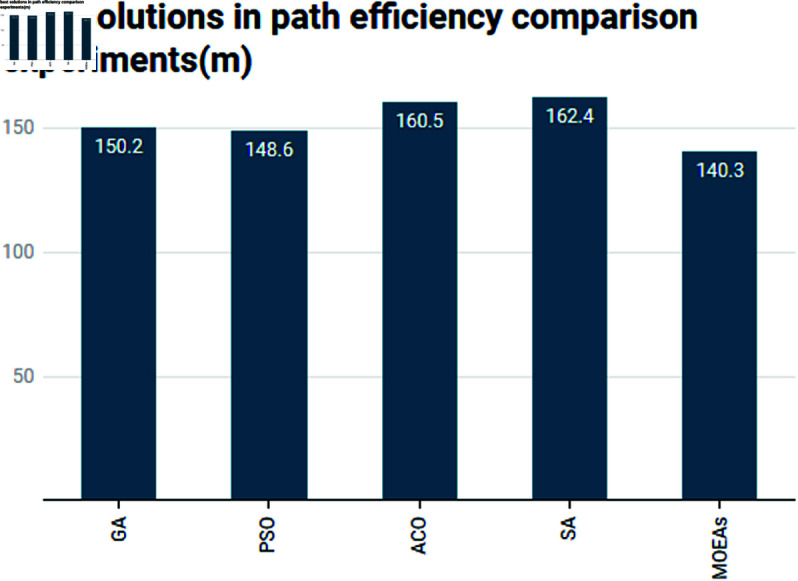
Comparison of best solutions in path efficiency comparison experiments.

In an area like Tongzhou, where population growth has led to crowded spaces, optimizing path efficiency is vital for reducing pedestrian congestion. As shown in Table 2, MOEAs provides the shortest paths, with the best solution being 140.3 m. This is particularly beneficial in urban spaces like Tongzhou, where the optimization of pedestrian flow directly correlates with the comfort and accessibility of green spaces (see Fig 6). PSO and GA also produce relatively efficient paths, but they fall short of the level achieved by MOEAs. ACO and SA, while effective at optimizing paths, result in longer paths, which may lead to less efficient pedestrian movement in highly populated areas. Thus, MOEAs emerges as the most suitable algorithm for path efficiency in dense urban environments like Tongzhou.

**Aesthetic Quality Analysis.** Aesthetic quality is essential in landscape design, particularly in areas like Tongzhou, where green spaces contribute to the overall urban environment and residents’ well-being. Aesthetic quality is evaluated based on expert opinions, with a rating scale from 1 to 10, where 10 represents the highest level of visual harmony and appeal:


$$f_{3}(x)=\text{Aesthetic Score (1--10)}$$

Table 4 provides the aesthetic quality scores for each algorithm.

**Table 4:** Aesthetic quality for different algorithms in Tongzhou district.

Algorithm	GA	PSO	ACO	SA	MOEAs
Best Solution	8.4	8.6	7.9	7.5	9.2
Average Solution	7.8	8.0	7.4	7.0	8.8
Worst Solution	7.1	7.3	6.8	6.5	8.2

**Fig 7:**
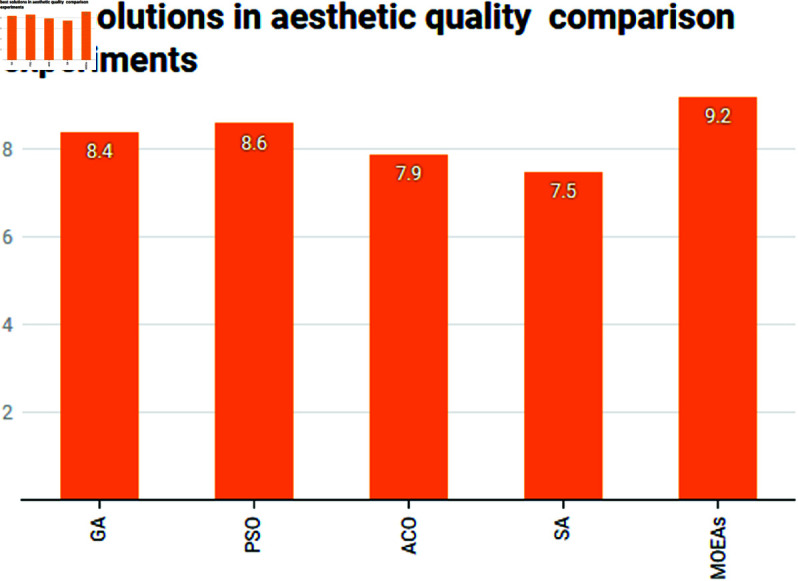
Comparison of best solutions in aesthetic quality comparison experiments.

In a rapidly developing area like Tongzhou, the aesthetic quality of green spaces plays a key role in enhancing the residents’ quality of life. As shown in Table 3, MOEAs achieves the highest aesthetic score, with the best solution at 9.2. This result indicates that MOEAs produces designs that not only meet functional requirements but also offer visually appealing and harmonious layouts (see Fig 7). PSO also achieves high aesthetic scores, with a best solution score of 8.6, reflecting its ability to maintain a balance between aesthetics and functionality. On the other hand, ACO and SA produce relatively lower aesthetic scores, with SA being the least effective in terms of visual quality. This reflects the nature of these algorithms, where ACO focuses more on path optimization, and SA tends to prioritize other objectives at the expense of aesthetic appeal. Therefore, MOEAs is the most appropriate algorithm for achieving high aesthetic quality in the landscape design of urban areas such as Tongzhou.

The analysis of the experimental results reveals that MOEAs outperforms other algorithms in all three key metrics: space utilization efficiency, path efficiency, and aesthetic quality. The results highlight the suitability of MOEAs for optimizing the landscape design in urban areas like Tongzhou, where factors such as population density and limited space require sophisticated solutions that balance multiple objectives. Although other algorithms like PSO and GA also perform well in specific areas, MOEAs provides the most comprehensive solution. ACO and SA, while effective in path optimization, are less suitable for maximizing space and aesthetic quality in such urban contexts. Overall, MOEAs emerges as the most balanced and effective optimization method for landscape garden green space design in Tongzhou District.

## 5 Conclusion and outlook

### 5.1 Conclusion

This study addresses the multi-objective optimization problem in landscape garden green space design, focusing on improving space utilization efficiency, path efficiency, and aesthetic quality. We propose a multi-objective evolutionary algorithm (MOEAs) as a solution to this problem, which is tailored for the urban environment of Tongzhou District, a rapidly developing area with high population density. The experimental results demonstrate that MOEAs outperforms other algorithms, such as GA, PSO, ACO, and SA, in all three objectives. Specifically, MOEAs achieved a space utilization efficiency of 90.2%, a path length of 140.3 m, and an aesthetic quality score of 9.2, outperforming the best results from GA (85.3%, 150.2 m, 8.4), PSO (88.5%, 148.6 m, 8.6), ACO (82.4%, 160.5 m, 7.9), and SA (80.1%, 162.4 m, 7.5). In conclusion, MOEAs provides the most comprehensive and efficient solution for optimizing landscape garden green space design in urban areas like Tongzhou, offering superior performance in balancing spatial efficiency, path optimization, and aesthetic quality.

### 5.2 Outlook

One limitation of this study is the dependency on the accuracy of the dataset used for the landscape design of the Tongzhou District green space. The current dataset is based on initial design concepts and demographic data, which may not fully capture the dynamic nature of urban expansion and potential future changes in population density and land use patterns. In future work, we plan to incorporate more comprehensive and up-to-date data sources, including geographic information systems (GIS) and real-time sensor data, to better reflect the real-time conditions of urban environments. Additionally, the current dataset does not account for seasonal variations, which could influence both the aesthetic quality and space utilization efficiency. By integrating time-based data, such as seasonal growth patterns of vegetation and variations in public use of green spaces, we aim to enhance the robustness of the optimization model. Another potential improvement could involve refining the decision-making model by incorporating user feedback and participatory design approaches, ensuring that the optimization process better aligns with the preferences and needs of the local community.

Another limitation of the current study is the computational complexity associated with multi-objective evolutionary algorithms (MOEAs). Although MOEAs provided the best performance in optimizing multiple objectives, the high computational cost, especially when dealing with large-scale urban green space designs, limits its practical application in real-world projects. The optimization process requires extensive iterations and considerable computational power, which may not be feasible in time-sensitive or resource-constrained situations. To address this issue, future research will focus on improving the efficiency of the algorithm by exploring hybrid approaches, such as combining MOEAs with reinforcement learning or metaheuristic techniques, to reduce computation time without compromising solution quality. Moreover, we plan to explore parallel computing methods and cloud-based architectures to distribute computational loads and improve the scalability of the model. Furthermore, incorporating machine learning techniques to predict the most promising areas of the design space could help in narrowing down the search space and significantly reduce the number of iterations required for optimization, making the algorithm more practical for large-scale real-world applications.
